# Supplementary figures and images for: Sedimentary Ancient DNA (sedaDNA) Reveals Fungal Diversity and Environmental Drivers of Community Changes throughout the Holocene in the Present Boreal Lake Lielais Svētiņu (Eastern Latvia)
Source: Microorganisms. 2021 Mar 31;9(4):719. doi: 10.3390/microorganisms9040719 (PMC8066534; doi:10.3390/microorganisms9040719)

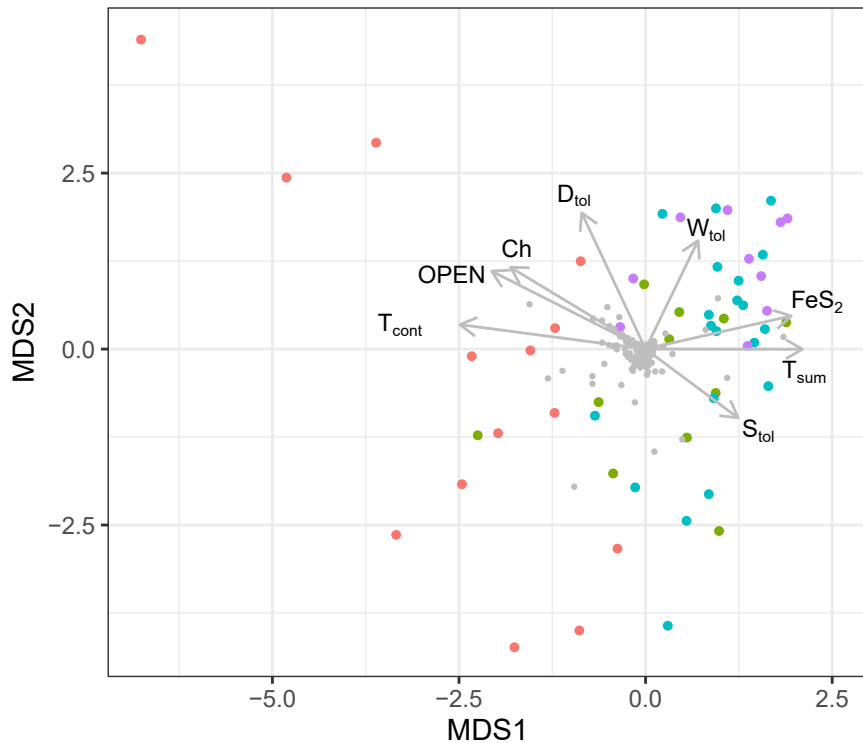

|            | r2   | p     |
|------------|------|-------|
| $T_{cont}$ | 0.39 | <0.01 |
| OPEN       | 0.34 | <0.01 |
| Ch         | 0.29 | <0.01 |
| $W_{tol}$  | 0.18 | <0.01 |
| $D_{tol}$  | 0.28 | <0.01 |
| $FeS_2$    | 0.25 | <0.01 |
| $T_{sum}$  | 0.28 | <0.01 |
| $S_{tol}$  | 0.16 | 0.011 |

Supplement: Supplementary file 1 [file microorganisms-09-00719-s001.zip › supplement failid/Figure S3.pdf]

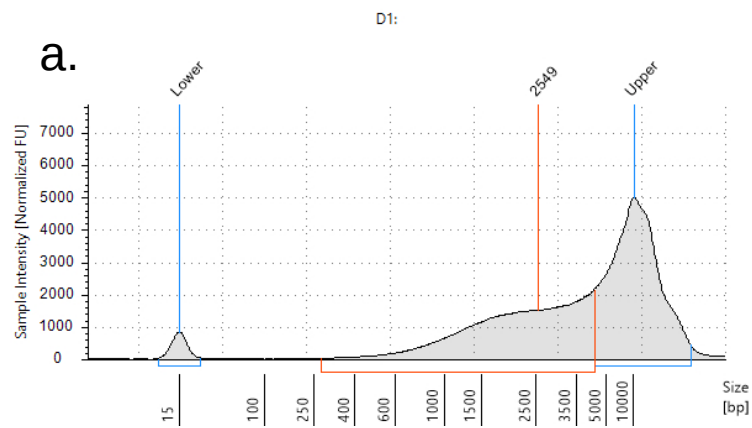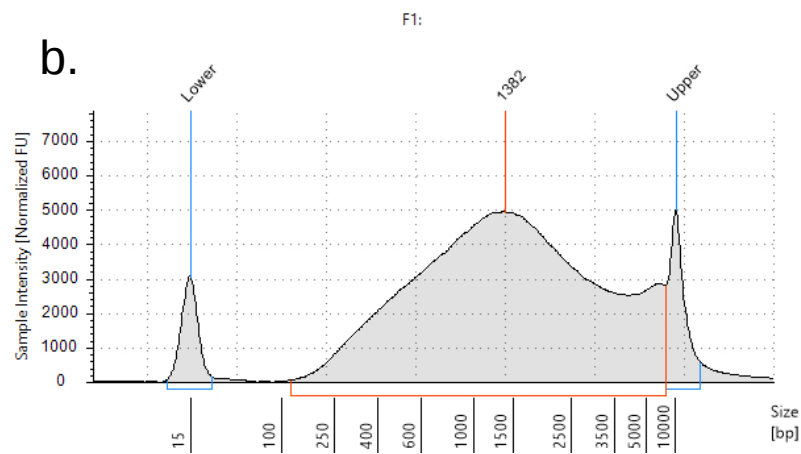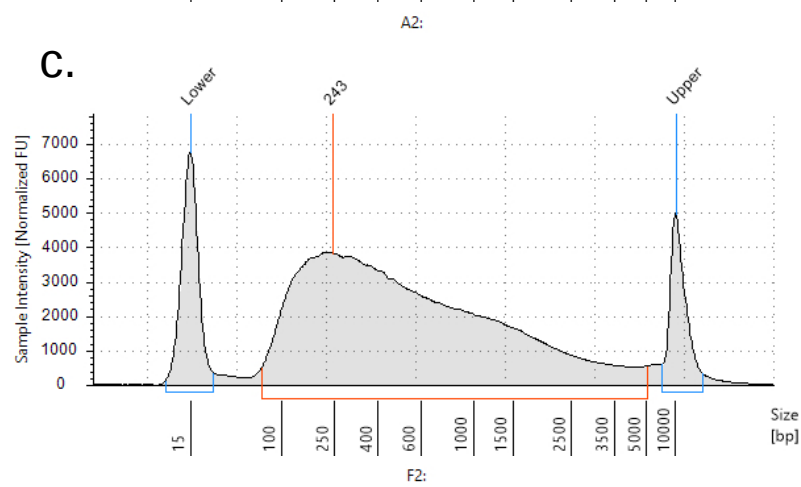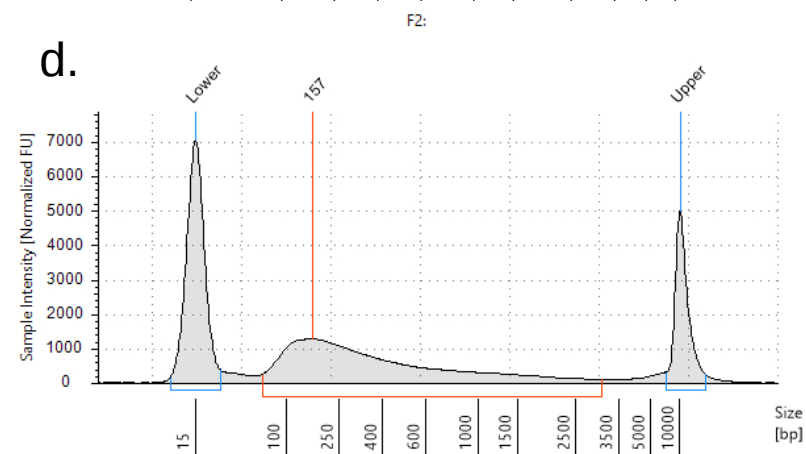

Supplement: Supplementary file 1 [file microorganisms-09-00719-s001.zip › supplement failid/Figure S1.pdf]

### Ascomycota

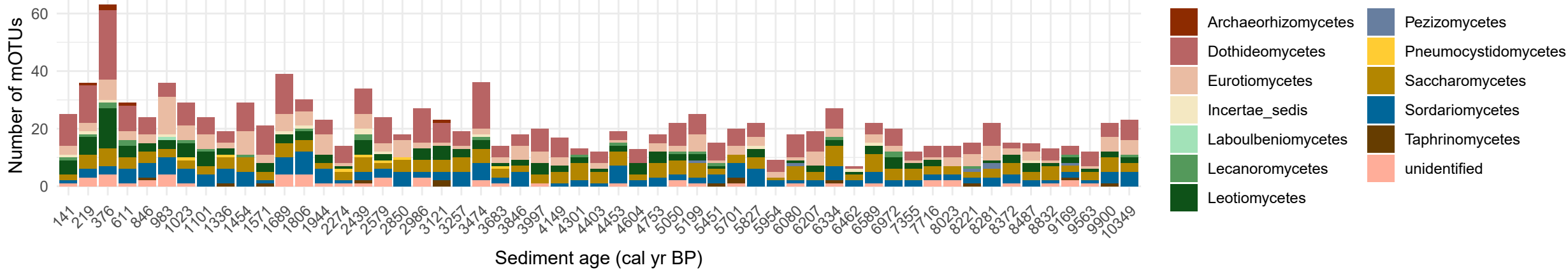

### Basidiomycota

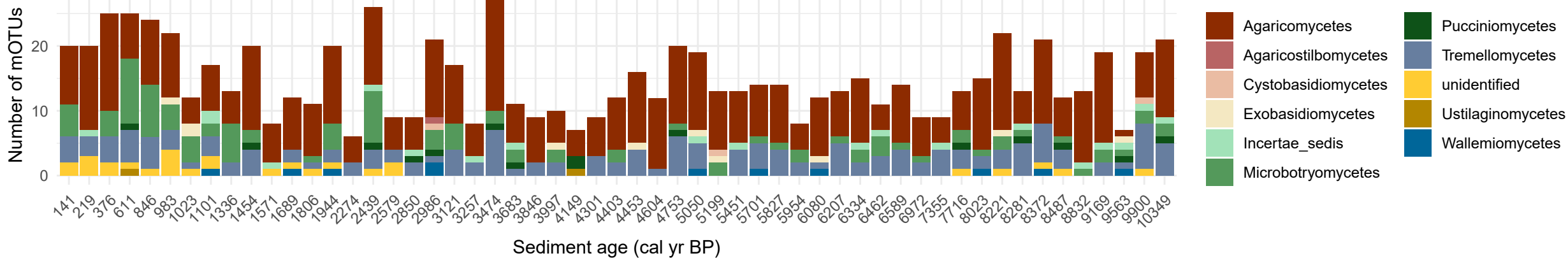

### Chytridiomycota

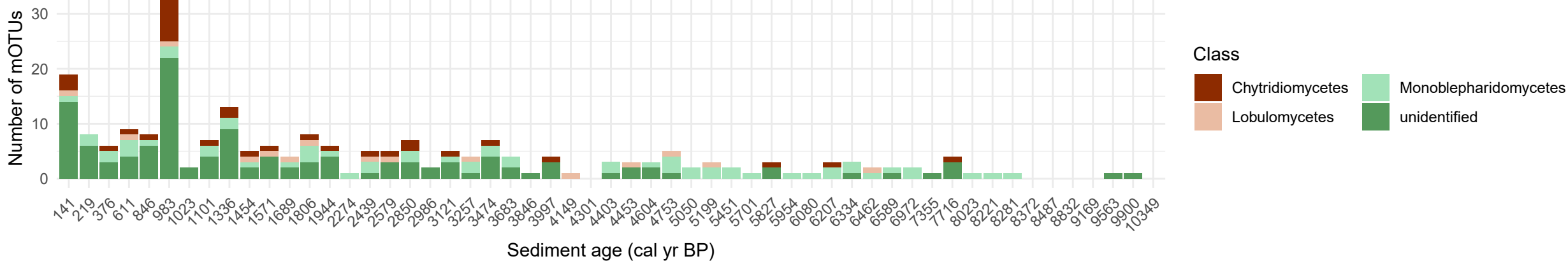

Supplement: Supplementary file 1 [file microorganisms-09-00719-s001.zip › supplement failid/Figure S2.pdf]

A)

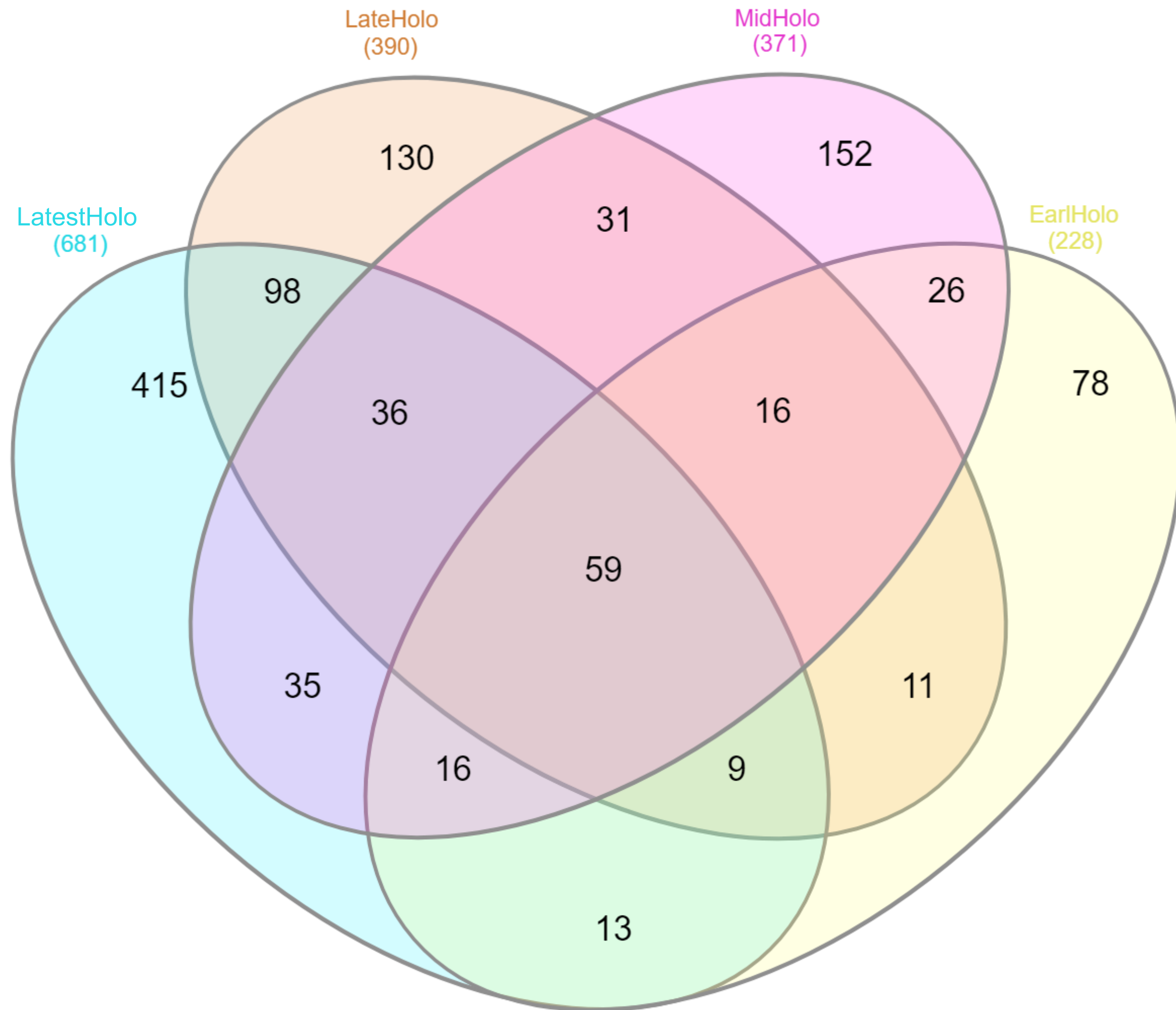

B)

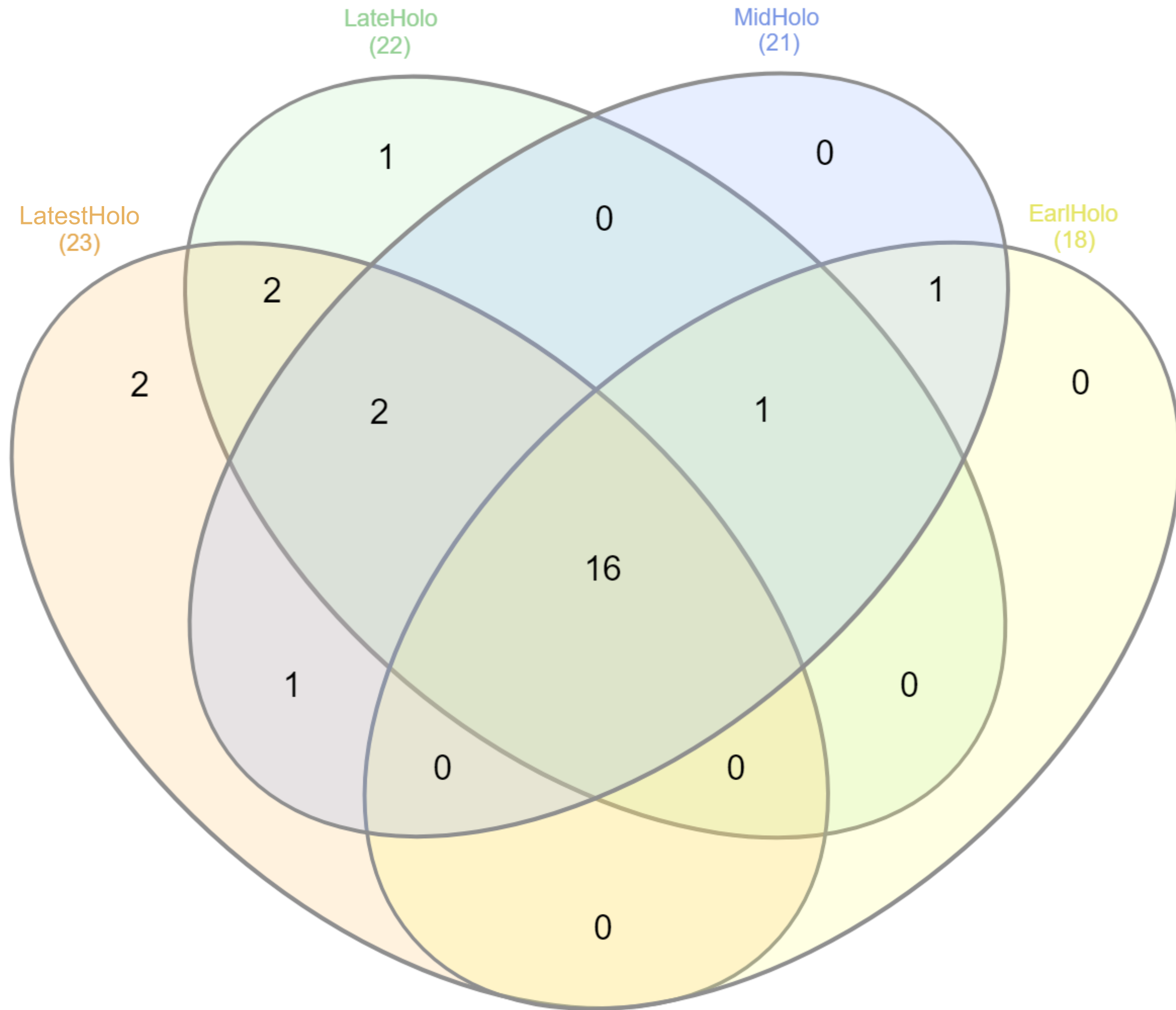

Supplement: Supplementary file 1 [file microorganisms-09-00719-s001.zip › supplement failid/Figure S4.pdf]

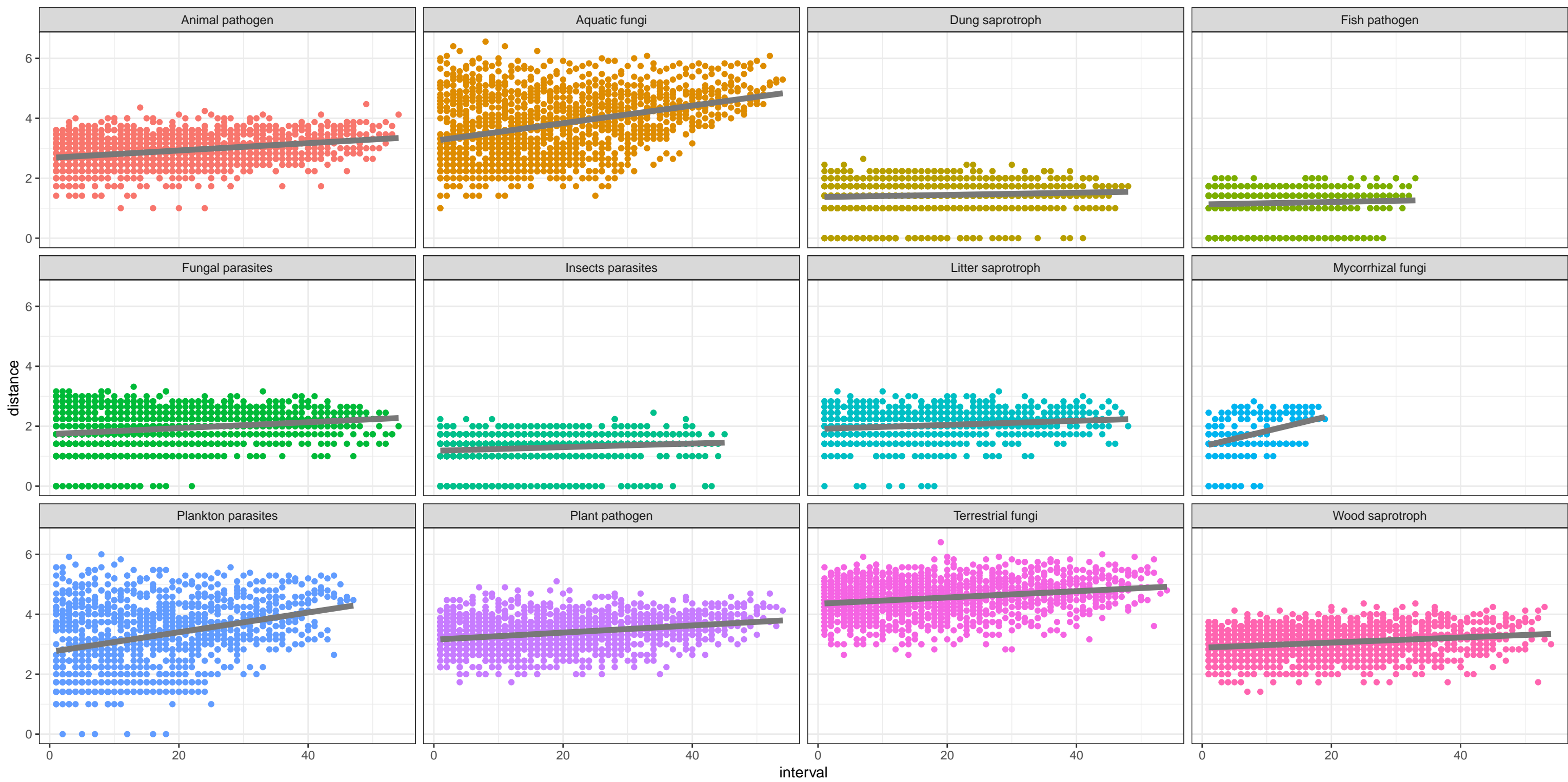

Supplement: Supplementary file 1 [file microorganisms-09-00719-s001.zip › supplement failid/Figure S5.pdf]

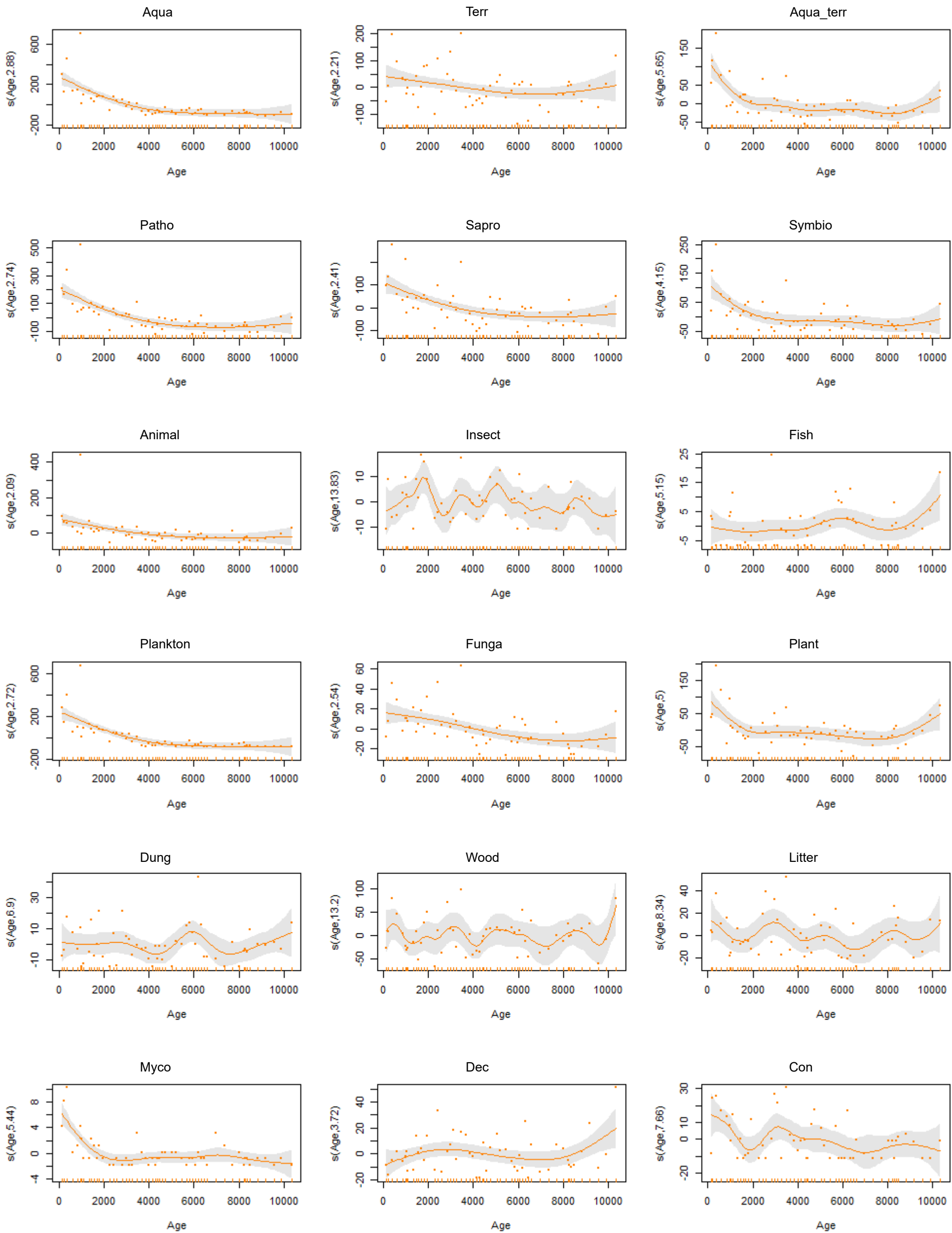

Supplement: Supplementary file 1 [file microorganisms-09-00719-s001.zip › supplement failid/Figure S6.pdf]
